# Supplementary material for: Amplifying the redistribution of somato-dendritic inhibition by the interplay of three interneuron types
Source: PLoS Comput Biol. 2019 May 16;15(5):e1006999. doi: 10.1371/journal.pcbi.1006999 (PMC6541306; doi:10.1371/journal.pcbi.1006999)
Supplement: S1 Fig — When one of the connections, VIP→SOM (w^SV) or SOM→VIP (w^VS), is kept constant, increasing the respective other weight leads to a strengthening of the amplification. Fixed weight was set to w^VS/SV=1. (PDF) [file pcbi.1006999.s001.pdf]

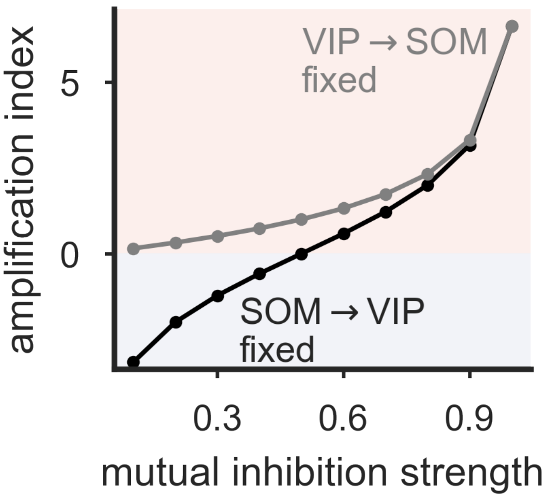

Fig S1. Asymmetric mutual inhibition strengths for SOM and VIP neurons also enhances the amplification index. When one of the connections, VIP→SOM ($\hat{\mathrm{w}}_{\mathrm{SV}}$) or SOM→VIP ($\hat{\mathrm{w}}_{\mathrm{VS}}$), is kept constant, increasing the respective other weight leads to a strengthening of the amplification. Fixed weight was set to $\hat{w}_{\mathrm{VS/SV}} = 1$.
