# Supplementary material for: Amplifying the redistribution of somato-dendritic inhibition by the interplay of three interneuron types
Source: PLoS Comput Biol. 2019 May 16;15(5):e1006999. doi: 10.1371/journal.pcbi.1006999 (PMC6541306; doi:10.1371/journal.pcbi.1006999)
Supplement: S2 Fig — When one of the recurrent connections, VIP→VIP (w^VV) or SOM→SOM (w^SS), is kept constant, increasing the respective other weight leads to a decrease of the amplification index. Fixed weight was set to w^SS/VV=0.5. Mutual inhibition strength w^=0.8. (PDF) [file pcbi.1006999.s002.pdf]

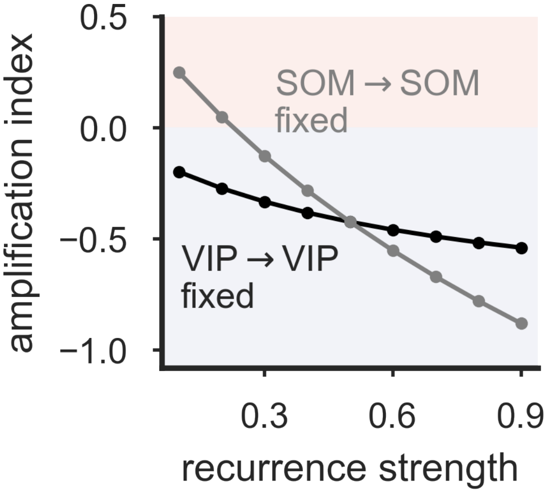

Fig S2. Asymmetric recurrence strengths for SOM and VIP neurons also reduce the amplification index. When one of the recurrent connections, VIP→VIP ($\hat{\mathrm{w}}_{\mathrm{VV}}$) or SOM→SOM ($\hat{\mathrm{w}}_{\mathrm{SS}}$), is kept constant, increasing the respective other weight leads to a decrease of the amplification index. Fixed weight was set to $\hat{\mathrm{w}}_{\mathrm{SS/VV}} = 0.5$. Mutual inhibition strength $\hat{w} = 0.8$.
