# Supplementary material for: Amplifying the redistribution of somato-dendritic inhibition by the interplay of three interneuron types
Source: PLoS Comput Biol. 2019 May 16;15(5):e1006999. doi: 10.1371/journal.pcbi.1006999 (PMC6541306; doi:10.1371/journal.pcbi.1006999)
Supplement: S3 Fig — Firing rate traces for SOM (blue) and VIP (green) neurons for a range of adaptation strengths (bS/V ∈ {0.4, 0.6, 0.8, 1}). Off-diagonal plots correspond to asymmetric adaptation strengths. Mutual inhibition strength w^=1.3, adaptation time constants τa = 50 ms. (PDF) [file pcbi.1006999.s003.pdf]

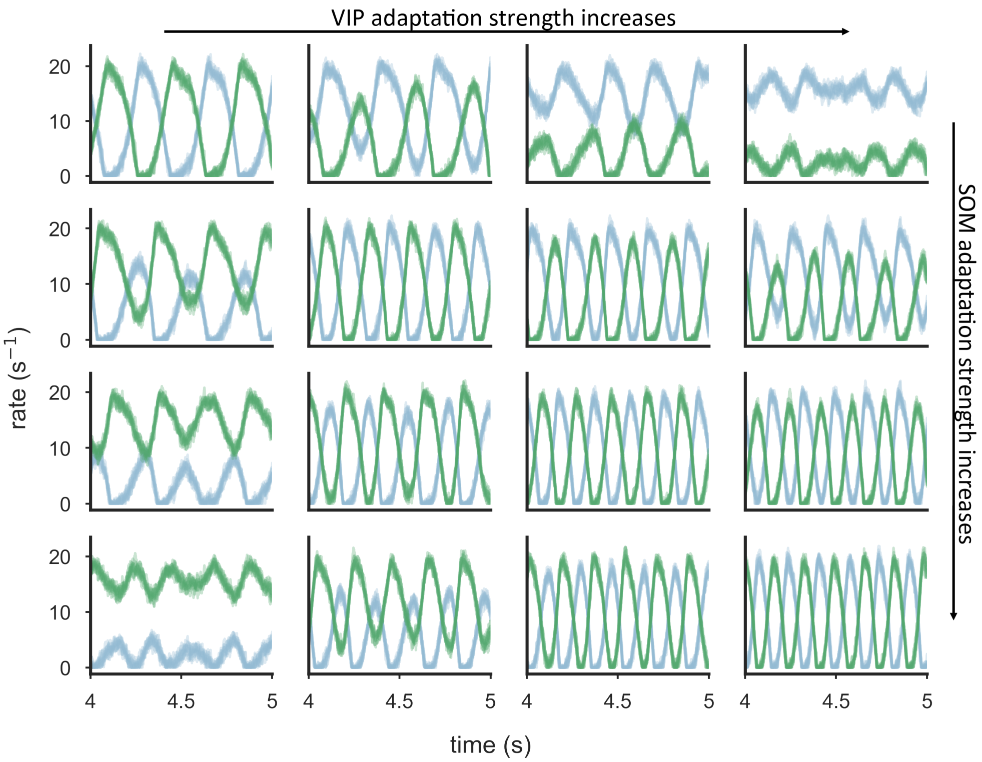

Fig S3. Oscillations also arise for asymmetric adaptation strengths in SOM and VIP neurons, with altered firing rate and oscillation frequency. Firing rate traces for SOM (blue) and VIP (green) neurons for a range of adaptation strengths ($b_{\mathrm{S/V}} \in \{0.4, 0.6, 0.8, 1\}$). Off-diagonal plots correspond to asymmetric adaptation strengths. Mutual inhibition strength $\hat{w} = 1.3$, adaptation time constants $\tau_a = 50$ ms.
