# Supplementary material for: Amplifying the redistribution of somato-dendritic inhibition by the interplay of three interneuron types
Source: PLoS Comput Biol. 2019 May 16;15(5):e1006999. doi: 10.1371/journal.pcbi.1006999 (PMC6541306; doi:10.1371/journal.pcbi.1006999)
Supplement: S4 Fig — Firing rate traces for SOM (blue) and VIP (green) neurons for a range of adaptation time constants (τa,S/V ∈ {50, 100, 200, 400} ms). Larger adaptation time constants cause longer active states. Mutual inhibition strength w^=1.3, Adaptation strength b = 0.5. (PDF) [file pcbi.1006999.s004.pdf]

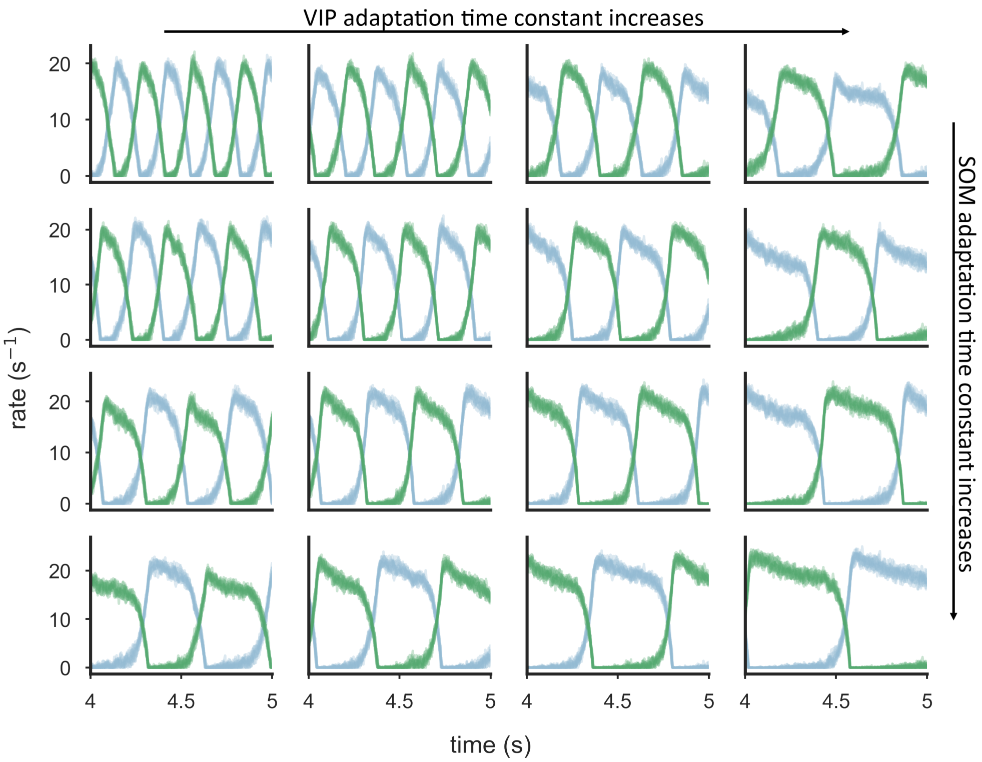

Fig S4. Asymmetric adaptation time constants for SOM and VIP neurons lead to different duration of active and inactive periods. Firing rate traces for SOM (blue) and VIP (green) neurons for a range of adaptation time constants ($\tau_{a,S/V} \in \{50, 100, 200, 400\}$ ms). Larger adaptation time constants cause longer active states. Mutual inhibition strength $\hat{w} = 1.3$, Adaptation strength $b = 0.5$.
