# Supplementary material for: Amplifying the redistribution of somato-dendritic inhibition by the interplay of three interneuron types
Source: PLoS Comput Biol. 2019 May 16;15(5):e1006999. doi: 10.1371/journal.pcbi.1006999 (PMC6541306; doi:10.1371/journal.pcbi.1006999)
Supplement: S5 Fig — Left: Bifurcation diagram reveals distinct operation modes: all interneurons are active (divided into amplification and attenuation regime), winner-take-all (WTA) regime leading to a switch, and two pathological states (WTA in each population separately and total WTA). Regime boundaries (black lines) are obtained from a mathematical analysis (see S1 Appendix). Right: Example firing rate traces for all SOM (blue) and VIP (green) neurons for four network settings (see markers) taken from the bifurcation diagram. NSOM = NVIP = 5. SOM and VIP neurons were stimulated with an input consisting of i) a constant component of 25/s and ii) individual noise drawn at each time t from a Gaussian distribution with zero mean and SD of 1/s. (PDF) [file pcbi.1006999.s005.pdf]

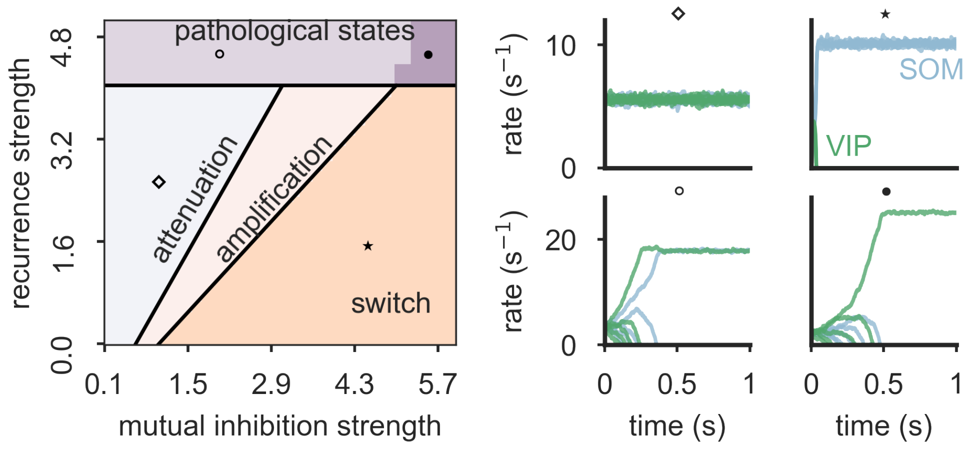

Fig S5. Dynamical states of the SOM-VIP motif with recurrence.
Left: Bifurcation diagram reveals distinct operation modes: all interneurons are active (divided into amplification and attenuation regime), winner-take-all (WTA) regime leading to a switch, and two pathological states (WTA in each population separately and total WTA). Regime boundaries (black lines) are obtained from a mathematical analysis (see Appendix). Right: Example firing rate traces for all SOM (blue) and VIP (green) neurons for four network settings (see markers) taken from the bifurcation diagram. $N_{\mathrm{SOM}} = N_{\mathrm{VIP}} = 5$. SOM and VIP neurons were stimulated with an input consisting of i) a constant component of $25/s$ and ii) individual noise drawn at each time t from a Gaussian distribution with zero mean and SD of $1/s$.
