# Supplementary material for: Amplifying the redistribution of somato-dendritic inhibition by the interplay of three interneuron types
Source: PLoS Comput Biol. 2019 May 16;15(5):e1006999. doi: 10.1371/journal.pcbi.1006999 (PMC6541306; doi:10.1371/journal.pcbi.1006999)
Supplement: S7 Fig — (A) Example phase planes (top) for three distinct values of the modulatory input (cf. bottom). The intersection points of SOM- (blue) and VIP-nullcline (green) correspond to the fixed points that are either stable (filled circle) or unstable (open circle). The vector field shows the direction and strength of flow. In a WTA regime, the network exhibits bistability for a range of modulatory input values, leading to hysteresis (bottom). (B) Same as above for the full microcircuit. PC rate exhibits two stable states for a range of modulatory inputs. The steady-state activity depends on the initial state. Parameter (A–B): Mutual inhibition strength w^=1.05. (PDF) [file pcbi.1006999.s007.pdf]

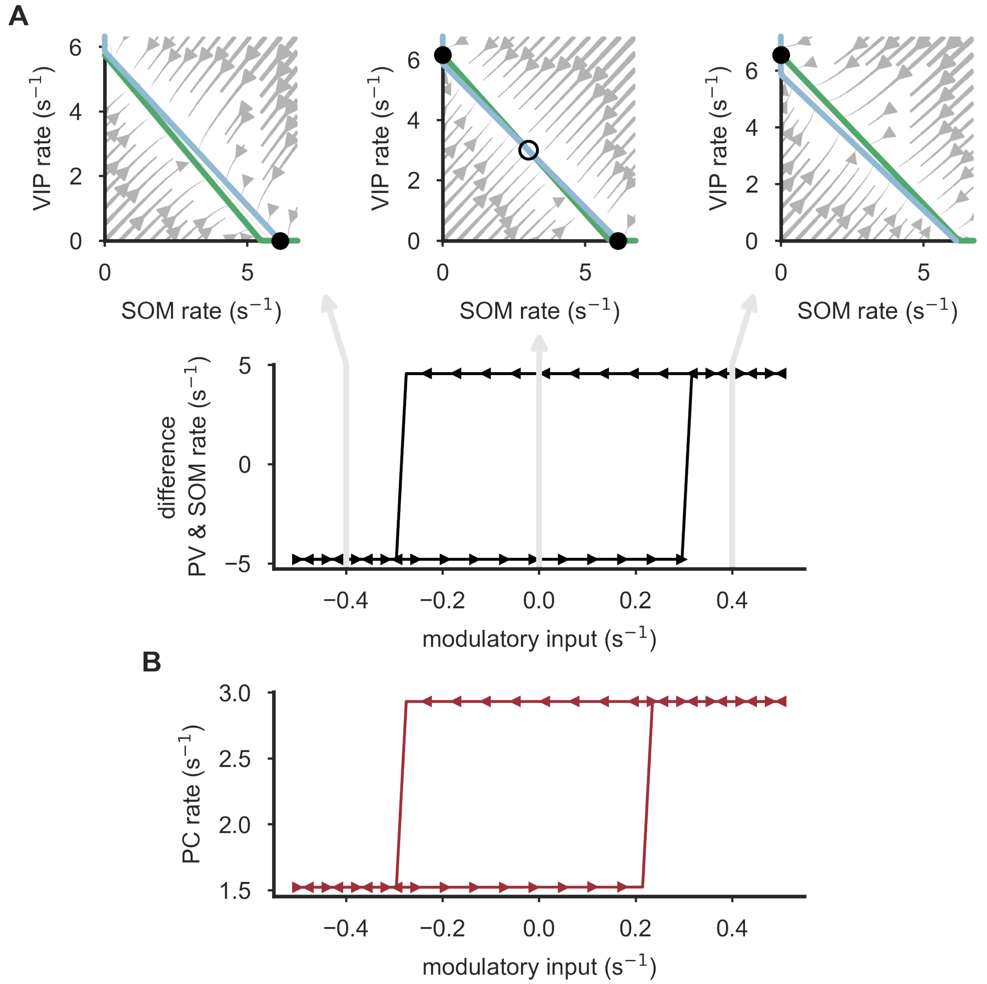

Fig S7. WTA between SOM and VIP neurons causes hysteresis in the reduced interneuron network and full microcircuit. (A) Example phase planes (top) for three distinct values of the modulatory input (cf. bottom). The intersection points of SOM- (blue) and VIP-nullcline (green) correspond to the fixed points that are either stable (filled circle) or unstable (open circle). The vector field shows the direction and strength of flow. In a WTA regime, the network exhibits bistability for a range of modulatory input values, leading to hysteresis (bottom). **(B)** Same as above for the full microcircuit. PC rate exhibits two stable states for a range of modulatory input. The steady-state activity depends on the initial state. Parameter (A - B): Mutual inhibition strength $\hat{w} = 1.05$.
