# Supplementary material for: Amplifying the redistribution of somato-dendritic inhibition by the interplay of three interneuron types
Source: PLoS Comput Biol. 2019 May 16;15(5):e1006999. doi: 10.1371/journal.pcbi.1006999 (PMC6541306; doi:10.1371/journal.pcbi.1006999)
Supplement: S1 Appendix — Mathematical analysis of a simplified model that comprises only SOM and VIP cell populations either with recurrent connections among both SOM and VIP neurons or adaptation. The derivations provide analytical conditions for the parameter boundaries between different computational states of the SOM-VIP motif. Approximations for these boundaries for the nonlinear case that includes short-term plasticity are also provided. (PDF) [file pcbi.1006999.s008.pdf]

S1 Appendix: Amplifying the redistribution of somato-dendritic inhibition by the interplay of three interneuron types

Loreen Hertäg[1,2], Henning Sprekeler[1,2],

1 Modelling of Cognitive Processes, Berlin Institute of Technology, Berlin, Germany
2 Bernstein Center for Computational Neuroscience, Berlin, Germany

* h.sprekeler@tu-berlin.de, loreen.hertaeg@tu-berlin.de

Mathematical analysis of the SOM-VIP motif

In order to characterize the computational repertoire of the SOM-VIP motif, we performed an extensive mathematical analysis of a simplified model that comprises only SOM and VIP cell populations. Moreover, we neglected the rectifying nonlinearity that ensures that firing rates remain positive. We will start with networks that take into account either recurrent connections among both SOM and VIP neurons or adaptation, but ignore short-term plasticity. In these cases, the circuits are described by linear dynamical systems and therefore allow detailed bifurcation analyses. The derivations provide analytical conditions for the parameter boundaries between different computational states of the SOM-VIP motif. We then continue to derive approximations for these boundaries for the nonlinear case that includes short-term plasticity.

Bifurcation analysis: Recurrent inhibition

We consider a network composed of SOM (hereafter only S) and VIP neurons (hereafter only V) that are mutually and fully connected. Furthermore, we assume that each population comprises n cells. The circuit dynamics can then be described by

$$\dot{\mathbf{r}} = W\mathbf{r} + \mathbf{s} \tag{1}$$

with $\mathbf{r}^{\mathrm{T}} = [r_{\mathrm{S}_1}, \dots, r_{\mathrm{S}_\mathrm{n}}, r_{\mathrm{V}_1}, \dots, r_{\mathrm{V}_\mathrm{n}}]$. $\mathbf{s}$ represents a vector of external stimuli and W is a block matrix of the form

$$W = \begin{bmatrix} U_{\mathrm{S}\leftarrow\mathrm{S}} & U_{\mathrm{S}\leftarrow\mathrm{V}} \\ U_{\mathrm{V}\leftarrow\mathrm{S}} & U_{\mathrm{V}\leftarrow\mathrm{V}}, \end{bmatrix} \tag{2}$$

which contains the connection weights. When recurrence is included , the $(n \times n)$ blocks have the following form

$$U_{\mathrm{S}\leftarrow\mathrm{S}} = \begin{bmatrix} -1/\tau_\mathrm{S} & -w_\mathrm{S}/\tau_\mathrm{S} & \dots & -w_\mathrm{S}/\tau_\mathrm{S} \\ -w_\mathrm{S}/\tau_\mathrm{S} & -1/\tau_\mathrm{S} & \dots & -w_\mathrm{S}/\tau_\mathrm{S} \\ \vdots & \vdots & \ddots & \vdots \\ -w_\mathrm{S}/\tau_\mathrm{S} & -w_\mathrm{S}/\tau_\mathrm{S} & \dots & -1/\tau_\mathrm{S} \end{bmatrix} \qquad U_{\mathrm{S}\leftarrow\mathrm{V}} = -\frac{w_{\mathrm{SV}}}{\tau_\mathrm{S}} J_{\mathrm{n,n}} \tag{3}$$

$$U_{\mathrm{V}\leftarrow\mathrm{V}} = \begin{bmatrix} -1/\tau_\mathrm{V} & -w_\mathrm{V}/\tau_\mathrm{V} & \dots & -w_\mathrm{V}/\tau_\mathrm{V} \\ -w_\mathrm{V}/\tau_\mathrm{V} & -1/\tau_\mathrm{V} & \dots & -w_\mathrm{V}/\tau_\mathrm{V} \\ \vdots & \vdots & \ddots & \vdots \\ -w_\mathrm{V}/\tau_\mathrm{V} & -w_\mathrm{V}/\tau_\mathrm{V} & \dots & -1/\tau_\mathrm{V} \end{bmatrix} \qquad U_{\mathrm{V}\leftarrow\mathrm{S}} = -\frac{w_{\mathrm{VS}}}{\tau_\mathrm{V}} J_{\mathrm{n,n}} \tag{4}$$

where $J_{\mathrm{n,n}}$ is a squared matrix of dimension n with all entries equal one. Note that autapses were not included and that we chose the convention that the weight parameters w_i are positive.

The dynamical properties of the circuit are described by its eigenvalues, which are given by the zero crossings of the characteristic polynomial of the matrix W,

$$0 = \det\left(\left(U_{\mathrm{S}\leftarrow\mathrm{S}} - \lambda\mathbb{1}\right)\left(U_{\mathrm{V}\leftarrow\mathrm{V}} - \lambda\mathbb{1}\right) - U_{\mathrm{S}\leftarrow\mathrm{V}}U_{\mathrm{V}\leftarrow\mathrm{S}}\right), \tag{5}$$

where $\mathbb{1}$ denotes the n-dimensional identity matrix. Resolving the determinant yields the following condition for the eigenvalues:

$$\begin{aligned} 0 = &\left[\left(\frac{1}{\tau_\mathrm{S}} + \lambda\right)\left(\frac{1}{\tau_\mathrm{V}} + \lambda\right) + \frac{w_\mathrm{S} w_\mathrm{V}}{\tau_\mathrm{S}\tau_\mathrm{V}} - \left(\frac{1}{\tau_\mathrm{S}} + \lambda\right)\frac{w_\mathrm{V}}{\tau_\mathrm{V}} - \left(\frac{1}{\tau_\mathrm{V}} + \lambda\right)\frac{w_\mathrm{S}}{\tau_\mathrm{S}}\right]^{n-1} \\ &\cdot\left[\frac{\hat{w}_\mathrm{S}\hat{w}_\mathrm{V}}{\tau_\mathrm{S}\tau_\mathrm{V}} + \left(\frac{1}{\tau_\mathrm{S}} + \lambda\right)\left(\frac{1}{\tau_\mathrm{V}} + \lambda\right) + \left(\frac{1}{\tau_\mathrm{S}} + \lambda\right)\frac{\hat{w}_\mathrm{V}}{\tau_\mathrm{V}} + \left(\frac{1}{\tau_\mathrm{V}} + \lambda\right)\frac{\hat{w}_\mathrm{S}}{\tau_\mathrm{S}} - \frac{\hat{w}_\mathrm{SV}\hat{w}_\mathrm{VS}}{\tau_\mathrm{S}\tau_\mathrm{V}}\right], \end{aligned} \tag{6}$$

with the total recurrence strengths $\hat{w}_{mn} = (n-1)\, w_{mn}$ and the total mutual inhibition strengths $\hat{w}_m = n\, w_m$. Consequently, the eigenvalues of the linear system are

$$\lambda_\mathrm{S} = -\frac{1}{\tau_\mathrm{S}} + \frac{\hat{w}_\mathrm{S}}{(n-1)\tau_\mathrm{S}}, \tag{7}$$

$$\lambda_\mathrm{V} = -\frac{1}{\tau_\mathrm{V}} + \frac{\hat{w}_\mathrm{V}}{(n-1)\tau_\mathrm{V}},$$

$$\lambda_{+/-} = -\frac{1}{2}\left(\frac{1+\hat{w}_\mathrm{S}}{\tau_\mathrm{S}} + \frac{1+\hat{w}_\mathrm{V}}{\tau_\mathrm{V}}\right) \tag{8}$$

$$\pm\frac{1}{2}\sqrt{\left[\left(\frac{1}{\tau_\mathrm{V}} - \frac{1}{\tau_\mathrm{S}}\right) + \left(\frac{\hat{w}_\mathrm{V}}{\tau_\mathrm{V}} - \frac{\hat{w}_\mathrm{S}}{\tau_\mathrm{S}}\right)\right]^2 + 4\frac{\hat{w}_\mathrm{SV}\hat{w}_\mathrm{VS}}{\tau_\mathrm{S}\tau_\mathrm{V}}}. \tag{9}$$

While the first and second eigenvalues λ_S, λ_V have an algebraic and geometric multiplicity of $(n-1)$ and describe the dynamics of rate inhomogeneities within the two populations, the eigenvalues λ_+ and λ_- have an algebraic and geometric multiplicity of one and determine the dynamics of the population rates of the two interneuron types. Because $\hat{w}_{SV}$ and $\hat{w}_{VS}$ are positive, all eigenvalues are real, suggesting the absence of oscillations. For the sake of simplicity, we now analyze a symmetric situation, in which $\tau_\mathrm{S} = \tau_\mathrm{V} = \tau$, $\hat{w}_\mathrm{S} = \hat{w}_\mathrm{V} = w_\mathrm{r}$ and $\hat{w}_\mathrm{SV} = \hat{w}_\mathrm{VS} = w$. By analyzing the sign and the nature (complex or real) of the eigenvalues, we can define five dynamical regimes:

(i) All interneurons can be active and operate in an attenuation regime (no competition, all eigenvalues negative, weak mutual inhibition),

(ii) All interneurons can be active and operate in an amplification regime (no competition, all eigenvalues negative, strong mutual inhibition),

(iii) WTA between SOM and VIP neuron population (either SOM or VIP cells win; $\lambda_{+/-} > 0$, $\lambda_{S/V} < 0$),

(iv) WTA in each population separately (one VIP and one SOM cell survive, $\lambda_{+/-} < 0$, $\lambda_{S/V} > 0$ positive),

(v) Total WTA (only one single neuron is active, all eigenvalues positive).

The transition between the attenuation and the amplification regime ((i) and (ii)) is determined by the condition that the amplification index (equation (24) in main text) is

equal to one (for the symmetric case $\hat{w}_{\mathrm{VV}} = \hat{w}_{\mathrm{SS}} = \hat{w}_r$ and $\hat{w}_{\mathrm{SV}} = \hat{w}_{\mathrm{VS}} = \hat{w}$). The transition to a WTA regime within each neuron population emerges when $\lambda_{\mathrm{S}} = \lambda_{\mathrm{V}} = \lambda \geq 0$, yielding the condition

$$w_{\mathrm{r}} \geq (n-1). \tag{10}$$

Moreover, the transition to a WTA regime between SOM and VIP neurons occurs when $\lambda_+ \geq 0$, which yields

$$\hat{w} \geq 1 + \hat{w}_{\mathrm{r}}. \tag{11}$$

Bifurcation analysis: Adaptation

We extend the rate dynamics (see equation (1)) with linear differential equations describing the evolution of an adaptation current in order to derive qualitative changes in the bifurcation structure when adaptation (instead of recurrent inhibition) is present. The $(4 \times n)$-dimensional state vector $\mathbf{r}$ now includes the adaptation variables and is given by

$\mathbf{r}^{\mathrm{T}} = [r_{\mathrm{S_1}}, a_{\mathrm{S_1}}, ..., r_{\mathrm{S_n}}, a_{\mathrm{S_n}}, r_{\mathrm{V_1}}, a_{\mathrm{V_1}}, ..., r_{\mathrm{V_n}}, a_{\mathrm{V_n}}]$. Again, the dynamical system can be written in the format of equations (1) and (2), but the matrices $U_{\mathrm{m}\leftarrow\mathrm{n}}$ $(m, n \in \{S, V\})$ now themselves become block matrices:

$$U_{\mathrm{S}\leftarrow\mathrm{S}} = \begin{bmatrix} M_0 & 0 & ... & 0 \\ 0 & M_0 & ... & 0 \\ \vdots & \vdots & \ddots & \vdots \\ 0 & 0 & ... & M_0 \end{bmatrix} \qquad U_{\mathrm{S}\leftarrow\mathrm{V}} = \begin{bmatrix} N_0 & N_0 & ... & N_0 \\ N_0 & N_0 & ... & N_0 \\ \vdots & \vdots & \ddots & \vdots \\ N_0 & N_0 & ... & N_0 \end{bmatrix} \tag{12}$$

$$U_{\mathrm{V}\leftarrow\mathrm{S}} = \begin{bmatrix} P_0 & P_0 & ... & P_0 \\ P_0 & P_0 & ... & P_0 \\ \vdots & \vdots & \ddots & \vdots \\ P_0 & P_0 & ... & P_0 \end{bmatrix} \qquad U_{\mathrm{V}\leftarrow\mathrm{V}} = \begin{bmatrix} Q_0 & 0 & ... & 0 \\ 0 & Q_0 & ... & 0 \\ \vdots & \vdots & \ddots & \vdots \\ 0 & 0 & ... & Q_0 \end{bmatrix}. \tag{13}$$

Here, M_0, N_0, P_0 and Q_0 are (2×2) matrices given by

$$M_0 = \begin{bmatrix} -1/\tau_{\mathrm{S}} & -1/\tau_{\mathrm{S}} \\ b_{\mathrm{S}}/\tau_{\mathrm{a,S}} & -1/\tau_{\mathrm{a,S}} \end{bmatrix} \qquad N_0 = \begin{bmatrix} -w_{\mathrm{SV}}/\tau_{\mathrm{S}} & 0 \\ 0 & 0 \end{bmatrix} \tag{14}$$

$$Q_0 = \begin{bmatrix} -1/\tau_{\mathrm{V}} & -1/\tau_{\mathrm{V}} \\ b_{\mathrm{V}}/\tau_{\mathrm{a,V}} & -1/\tau_{\mathrm{a,V}} \end{bmatrix} \qquad P_0 = \begin{bmatrix} -w_{\mathrm{VS}}/\tau_{\mathrm{V}} & 0 \\ 0 & 0 \end{bmatrix} \tag{15}$$

with the adaptation strengths b_{S} and b_{V}, as well as adaptation time constants $\tau_{\mathrm{a,S}}$ and $\tau_{\mathrm{a,V}}$. As the block matrices do not commute, the eigenvalue condition for the characteristic polynomial is now given by [1]

$$\begin{aligned} 0 &= \det(W - \lambda \mathbb{1}) = \det(U_{\mathrm{S}\leftarrow\mathrm{S}} - \lambda \mathbb{1}) \\ &\quad \cdot \det([U_{\mathrm{V}\leftarrow\mathrm{V}} - \lambda \mathbb{1}] - U_{\mathrm{V}\leftarrow\mathrm{S}} \, [U_{\mathrm{S}\leftarrow\mathrm{S}} - \lambda \mathbb{1}]^{-1} \, U_{\mathrm{S}\leftarrow\mathrm{V}}). \end{aligned} \tag{16}$$

After some straightforward linear algebra, the eigenvalues are given by the zeros of the following equation:

$$\begin{aligned} 0 = &\left[\left(\frac{1}{\tau_{\mathrm{S}}} + \lambda\right)\left(\frac{1}{\tau_{\mathrm{a,S}}} + \lambda\right) + \frac{b_{\mathrm{S}}}{\tau_{\mathrm{S}}\tau_{\mathrm{a,S}}}\right]^{(n-1)} \left[\left(\frac{1}{\tau_{\mathrm{V}}} + \lambda\right)\left(\frac{1}{\tau_{\mathrm{a,V}}} + \lambda\right) + \frac{b_{\mathrm{V}}}{\tau_{\mathrm{V}}\tau_{\mathrm{a,V}}}\right]^{(n-1)} \\ &\cdot \left[\left\{\left(\frac{1}{\tau_{\mathrm{S}}} + \lambda\right)\left(\frac{1}{\tau_{\mathrm{a,S}}} + \lambda\right) + \frac{b_{\mathrm{S}}}{\tau_{\mathrm{S}}\tau_{\mathrm{a,S}}}\right\}\left\{\left(\frac{1}{\tau_{\mathrm{V}}} + \lambda\right)\left(\frac{1}{\tau_{\mathrm{a,V}}} + \lambda\right) + \frac{b_{\mathrm{V}}}{\tau_{\mathrm{V}}\tau_{\mathrm{a,V}}}\right\} \right. \\ &\left. - \frac{\hat{w}_{\mathrm{SV}}\hat{w}_{\mathrm{VS}}}{\tau_{\mathrm{S}}\tau_{\mathrm{V}}}\left(\frac{1}{\tau_{\mathrm{a,S}}} + \lambda\right)\left(\frac{1}{\tau_{\mathrm{a,V}}} + \lambda\right)\right]. \end{aligned} \tag{17}$$

For the sake of simplicity and comparability, we again consider the symmetric case $\tau_S = \tau_V = \tau$, $w_{SV} = w_{VS} = w$, $b_S = b_V = b$ and $\tau_{a,S} = \tau_{a,V} = \tau_a$. This simplifies the derivation of the eigenvalues considerably. We obtain three pairs of eigenvalues

$$\lambda_{1,\pm} = \frac{1}{2}\left[-\left(\frac{1}{\tau}+\frac{1}{\tau_a}\right) \pm \sqrt{\left(\frac{1}{\tau_a}-\frac{1}{\tau}\right)^2 - \frac{4\,b}{\tau\tau_a}}\right],$$
$$\lambda_{2,\pm} = \frac{1}{2}\left[-\left(\frac{1}{\tau}+\frac{1}{\tau_a}-\frac{\hat{w}}{\tau}\right) \pm \sqrt{\left(\frac{1}{\tau}-\frac{1}{\tau_a}-\frac{\hat{w}}{\tau}\right)^2 - \frac{4\,b}{\tau\tau_a}}\right],$$
$$\lambda_{3,\pm} = \frac{1}{2}\left[-\left(\frac{1}{\tau}+\frac{1}{\tau_a}+\frac{\hat{w}}{\tau}\right) \pm \sqrt{\left(\frac{1}{\tau}-\frac{1}{\tau_a}+\frac{\hat{w}}{\tau}\right)^2 - \frac{4\,b}{\tau\tau_a}}\right]. \quad (18)$$

The first two eigenvalues ($\lambda_{1,+}$ and $\lambda_{1,-}$) have both algebraic and geometric multiplicity of $(2n-2)$ and define the rate and adaptation dynamics of rate inhomogeneities within the two interneuron populations. Both eigenvalues are strictly negative for all parameters possible. Hence, in contrast to recurrence, adaptation does not lead to pathological states in which a single cell within one population silences the others (see conditions (iv) and (v) in section above). The last four eigenvalues have algebraic and geometric multiplicity of one and describe the interaction between the two populations. It can be easily seen that $\mathrm{Re}(\lambda_{3,\pm}) < 0$, so that instabilities – like WTA regimes – can only arise from the two eigenvalues $\lambda_{2,\pm}$. Depending on the sign and nature of these eigenvalues, we find four dynamical regimes:

(i) All interneurons can be active and operate in an attenuation regime (all eigenvalues real and negative, weak mutual inhibition),

(ii) All interneurons can be active and operate in an amplification regime (all eigenvalues real and negative, strong mutual inhibition),

(iii) WTA between SOM and VIP neuron population, described by the condition

$$b \leq \hat{w} - 1, \quad (19)$$

(iv) An oscillatory WTA regime (osc WTA) in which SOM and VIP neurons alternate between active and inactive states. It depends on two conditions

$$b > \hat{w} - 1,$$
$$\hat{w} > 1 + \tau/\tau_a. \quad (20)$$

The derivation of the amplification index A for a network with adaptation is analogous to the derivation with recurrent inhibition, and merely requires to replace the total recurrence strength $\hat{w}_r$ by the strength b of adaptation. Hence, the transition between the attenuation and the amplification regime ((i) and (ii)) is determined by this minor modification of equation (24) (see main text).

Strictly speaking, the last regime (iv) has to be separated into two subregimes, in which the two eigenvalues $\lambda_{2,\pm}$ either (a) form a complex conjugate pair with positive real part or (b) are both real and positive. In the former regime, an approximation of the oscillation frequency f can be obtained from the imaginary part of the eigenvalues:

$$f = \frac{1}{4\pi}\sqrt{\frac{4b}{\tau\tau_a} - \left(\frac{1}{\tau}-\frac{1}{\tau_a}-\frac{\hat{w}}{\tau}\right)^2}. \quad (21)$$

The oscillation frequency follows a square root function in b with a scaling factor determined by τ_{a}, and an offset controlled by both $\hat{w}$ and τ_{a}. The parameter regime (a), in which an approximation for the oscillation frequency can be obtained from a linear analysis is limited by the condition

$$b > \frac{\tau\tau_{\mathrm{a}}}{4}\left(\frac{1}{\tau} - \frac{1}{\tau_{\mathrm{a}}} - \frac{\hat{w}}{\tau}\right)^2 . \tag{22}$$

In the second regime (b), the circuit nevertheless displays oscillations when simulated. These rely on the rectifying nonlinearity for the firing rates, so their frequency cannot be calculated within the present linear analysis.

Bifurcation analysis: Adaptation and short-term facilitation

When synaptic adaptation (STF) is included in the model, the dynamical system equations become nonlinear and mathematically intractable. Thus, we can only derive approximate expressions for the transitions between the computational regimes (see (i) to (iv) in section above).

In zero-order approximation, we assume that the facilitation variable u_{ij} can be replaced by its steady-state value u_∞, for which we derive an expression below. The boundaries given by equations (19) and (20) can then be replaced by

$$b = u_\infty \hat{w} - 1, \tag{23}$$

$$u_\infty \hat{w} = 1 + \frac{\tau}{\tau_{\mathrm{a}}}. \tag{24}$$

These approximations allow to deduce the qualitative changes in the transition boundaries. As $u_\infty \geq 1$, the slope of equation (23) increases, leading to an enlarged WTA regime (i.e., switch-like state). Moreover, the transition to an oscillatory switch is pushed towards smaller mutual inhibition strengths and requires stronger adaptation (cf. equations (23)-(24)).

To qualify the regime boundaries, we have to derive an expression for the steady-state value u_∞ as a function of the parameters b and $\hat{w}$. To this end, we identify a self-consistent solution of the equations for u_∞ and the steady state of the population rates of the two interneuron types.

The steady state of the population rates $r_{\mathrm{V/S}} := \frac{1}{N_{\mathrm{V/S}}} \sum_i r_{\mathrm{V/S},i}$ is given by the conditions

$$r_{\mathrm{V}} = \bar{s} - \hat{w}\, u_\infty^S\, r_{\mathrm{S}} - b\, r_{\mathrm{V}}, \tag{25}$$

$$r_{\mathrm{S}} = \bar{s} - \hat{w}\, u_\infty^V\, r_{\mathrm{V}} - b\, r_{\mathrm{S}}. \tag{26}$$

Here, $\bar{s}$ denotes the averaged input. u_∞^S and u_∞^V represent the steady-state facilitation variable for SOM→VIP and VIP→SOM, respectively, which are given by

$$u_\infty^{\mathrm{V/S}} = \frac{1 + \tau_{\mathrm{f}} r_{\mathrm{V/S}}}{1 + U_{\mathrm{s}} \tau_{\mathrm{f}} r_{\mathrm{V/S}}}. \tag{27}$$

To express u_∞ in terms of b and $\hat{w}$, we have to resolve equation (26) for the population rates and insert these expressions into equation (27). Because the resulting equations are lengthy and non-informative, we consider two approximations. In the non-WTA regime, as intrinsic and synaptic parameters as well as the external stimulation are taken to be equal, we assume that the population firing rates of SOM and VIP neurons

are approximately equal on average $r_{\mathrm{V}} \approx r_{\mathrm{S}} \equiv r$. This leads to the following expression for the population rates r in terms of the system parameters:

$$r \overset{(25)}{\underset{(27)}{=}} \frac{\tau_{\mathrm{f}} U_{\mathrm{s}} \bar{s} - 1 - b - \hat{w} + \sqrt{(\tau_{\mathrm{f}} U_{\mathrm{s}} \bar{s} - 1 - b - \hat{w})^2 + 4 \bar{s} \tau_f \left[(1+b) U_{\mathrm{s}} + \hat{w}\right]}}{2 \tau_{\mathrm{f}} \left[(1+b) U_{\mathrm{s}} + \hat{w}\right]}. \tag{28}$$

By inserting equations (27) and (28) into equation (24), we obtained a good approximation of the transition boundary between the amplification regimes and the oscillatory switch state.

For the transition to a WTA regime, the assumption of equal rates is violated. To derive an expression for this transition boundary, we therefore replace the assumption of equal rates by an assumption of a WTA state, in which one of the rates is zero. Because all parameters are assumed to be symmetric, we can assume without loss of generality that the VIP population is the winner. We can then obtain conditions for the boundary by setting $r_S = 0$ in equations (25) and (26) and inserting equation (27) into equation (26):

$$\begin{aligned} r_{\mathrm{V}} &\overset{(26)}{\underset{(27)}{=}} \frac{\tau_{\mathrm{f}} U_{\mathrm{s}} \bar{s} - \hat{w} + \sqrt{(\tau_{\mathrm{f}} U_{\mathrm{s}} \bar{s} - \hat{w})^2 + 4 \hat{w} \tau_{\mathrm{f}} \bar{s}}}{2 \hat{w} \tau_{\mathrm{f}}}, \\ &\overset{(25)}{\underset{(27)}{=}} \frac{\bar{s}}{1+b}. \end{aligned} \tag{29}$$

Solving this equation for the adaptation strength b, we obtain

$$b = \frac{2 \hat{w} \tau_{\mathrm{f}} \bar{s}}{\tau_{\mathrm{f}} U_{\mathrm{s}} \bar{s} - \hat{w} + \sqrt{(\tau_{\mathrm{f}} U_{\mathrm{s}} \bar{s} - \hat{w})^2 + 4 \hat{w} \tau_{\mathrm{f}} \bar{s}}} - 1. \tag{30}$$

This approximation predicts the transition to the WTA regime qualitatively and quantitatively very well.

For deriving the transition boundary between attenuation and amplification, we note that the amplification threshold $A = 1$ is satisfied when the slopes for the full network and the reference network are equal,

$$\frac{d r_{\mathrm{S}}^{\mathrm{full}}}{d x_{\mathrm{mod}}} = \frac{d r_{\mathrm{S}}^{\mathrm{ref}}}{d x_{\mathrm{mod}}}. \tag{31}$$

The steady state equation for the population rates of the SOM neurons in the reference and full network, respectively, are given by

$$(1+b)\, r_{\mathrm{S}}^{\mathrm{ref}} = \bar{s} - x_{\mathrm{mod}}, \tag{32}$$

$$(1+b)\, r_{\mathrm{S}}^{\mathrm{full}} = \bar{s} - u_{\infty}^{V} r_{\mathrm{V}}, \tag{33}$$

where u_{∞}^{V} is given in equation (27). The population rate r_{V} of the VIP neurons in the full network obeys

$$(1+b)\, r_{\mathrm{V}} = \bar{s} + x_{\mathrm{mod}} - u_{\infty}^{S} r_{\mathrm{S}}^{\mathrm{full}}. \tag{34}$$

Taking the derivative of the steady-state equations (32) and (33) for full and reference network with respect to x_{mod} yields

$$\frac{d r_{\mathrm{S}}^{\mathrm{ref}}}{d x_{\mathrm{mod}}} = -\frac{1}{1+b}, \tag{35}$$

$$\frac{d r_{\mathrm{S}}^{\mathrm{full}}}{d x_{\mathrm{mod}}} = -\frac{w}{1+b} \frac{(1 + 2\tau_{\mathrm{f}} r_{\mathrm{V}})(1 + U_{\mathrm{s}} \tau_{\mathrm{f}} r_{\mathrm{V}}) - (1 + \tau_{\mathrm{f}} r_{\mathrm{V}}) U_{\mathrm{s}} \tau_{\mathrm{f}} r_{\mathrm{V}}}{(1 + U_{\mathrm{s}} \tau_{\mathrm{f}} r_{\mathrm{V}})^2} \frac{d r_{\mathrm{V}}}{d x_{\mathrm{mod}}}. \tag{36}$$

By taking the derivative of equation (34), we can express $\frac{dr_{\mathrm{V}}}{dx_{\mathrm{mod}}}$ as a function of $\frac{dr_{\mathrm{S}}^{\mathrm{full}}}{dx_{\mathrm{mod}}}$ and $r_{\mathrm{S}}^{\mathrm{full}}$. Furthermore, we assume again that $r_{\mathrm{V}} \approx r_{\mathrm{S}} \equiv r$ (cf. equation (28)). Solving for $\frac{dr_{\mathrm{S}}^{\mathrm{full}}}{dx_{\mathrm{mod}}}$, the condition (31) finally yields

$$(1+b)^2 - \hat{w}^2\gamma^2 \overset{(31)}{=} \hat{w}\gamma(1+b), \tag{37}$$

with

$$\gamma = \frac{(1+2\tau_{\mathrm{f}}r)(1+U_{\mathrm{s}}\tau_{\mathrm{f}}r) - (1+\tau_{\mathrm{f}}r)U_{\mathrm{s}}\tau_{\mathrm{f}}r}{(1+U_{\mathrm{s}}\tau_{\mathrm{f}}r)^2}. \tag{38}$$

The combination of equations (37), (38) and (28) provide an accurate prediction of the amplification threshold when STF and adaptation are present.

References

1. Silvester, J. R. Determinants of block matrices. *The Mathematical Gazette* **84**, 460–467 (2000).

2. Murayama, M. *et al.* Dendritic encoding of sensory stimuli controlled by deep cortical interneurons. *Nature* **457**, 1137 (2009).
