# Supplementary material for: Amplifying the redistribution of somato-dendritic inhibition by the interplay of three interneuron types
Source: PLoS Comput Biol. 2019 May 16;15(5):e1006999. doi: 10.1371/journal.pcbi.1006999 (PMC6541306; doi:10.1371/journal.pcbi.1006999)
Supplement: S6 Fig — (A) PCs in the full microcircuit (left) exhibit a steeper firing rate slope with increasing mutual inhibition strength (w^=0.7 and 0.9), increasing short-term facilitation (STF) (Us = 0.4 and 0.25, τf = 200 ms), but a reduced slope for increasing recurrence strength (w^r=0 and 0.3) and adaptation (b = 0.2 and 0.5, τa = 100 ms). (B) With adaptation, the frequency response analysis reveals a frequency-selective amplification. (C) Mean SOM-SOM neuron correlation is more sensitive to increasing recurrence strength than to adaptation strength (right). Parameter (B–C): Mutual inhibition strength w^=0.8. (PDF) [file pcbi.1006999.s006.pdf]

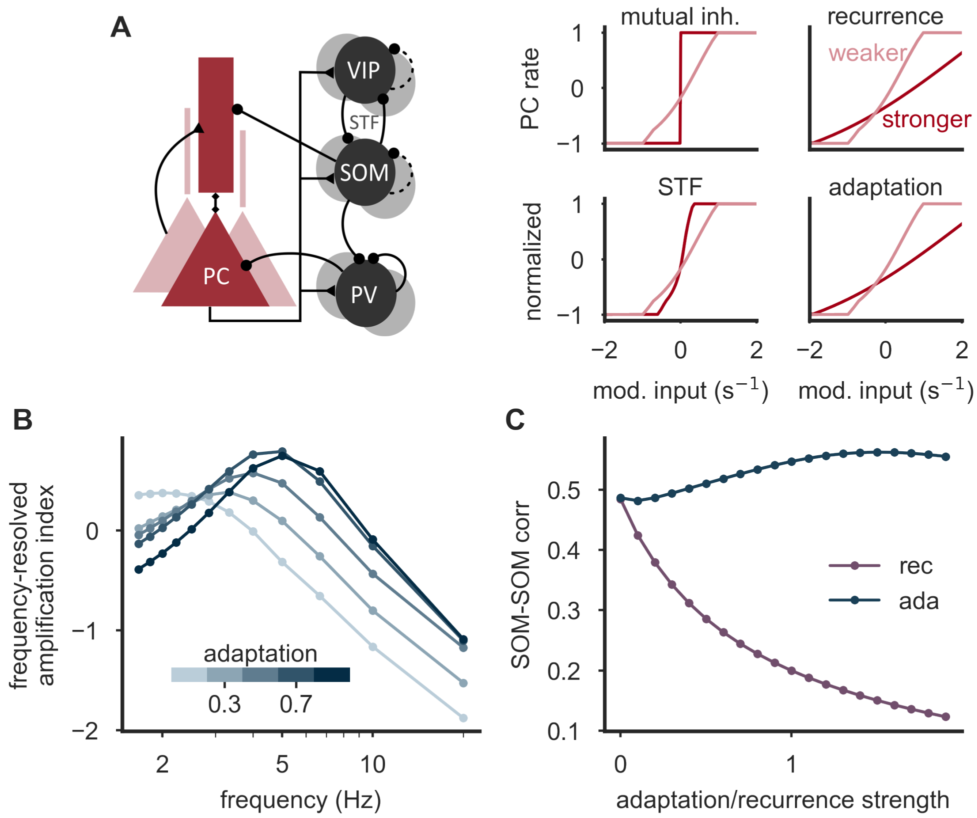

Fig S6. The full microcircuit with excitatory PC and inhibitory PV, SOM and VIP neurons shows the same phenomena as the reduced interneuron network. **(A)** PCs in the full microcircuit (left) exhibit a steeper firing rate slope with increasing mutual inhibition strength ($\hat{w} = 0.7$ and 0.9), increasing short-term facilitation (STF) ($U_{\mathrm{s}} = 0.4$ and 0.25, $\tau_{\mathrm{f}} = 200$ ms), but a reduced slope for increasing recurrence strength ($\hat{w}_r = 0$ and 0.3) and adaptation ($b = 0.2$ and 0.5, $\tau_a = 100$ ms). **(B)** With adaptation, the frequency response analysis reveals a frequency-selective amplification. **(C)** Mean SOM-SOM neuron correlation is more sensitive to increasing recurrence strength than to adaptation strength (right). Parameter (B - C): Mutual inhibition strength $\hat{w} = 0.8$.
